# Xylem K^+^ loading modulates K^+^ and Cs^+^ absorption and distribution in Arabidopsis under K^+^-limited conditions

**DOI:** 10.3389/fpls.2023.1040118

**Published:** 2023-09-22

**Authors:** Satomi Kanno, Ludovic Martin, Natacha Vallier, Serge Chiarenza, Tatsuya Nobori, Jun Furukawa, Laurent Nussaume, Alain Vavasseur, Nathalie Leonhardt

**Affiliations:** ^1^ AixMarseille University, French Alternative Energies and Atomic Energy Commission (CEA), National Center for Scientific Research (CNRS), Bioscience and Biotechnology Institute of Aix-Marseille (BIAM), Saint-Paul Lez Durance, France; ^2^ Faculty of Life and Environmental Sciences University of Tsukuba, Tsukuba, Ibaraki, Japan; ^3^ Institute for Advanced Research, Nagoya University, Nagoya, Japan; ^4^ Center for Research in Isotopes and Environmental Dynamics, University of Tsukuba, Tsukuba, Ibaraki, Japan

**Keywords:** *Arabidopsis thaliana*, HAK5, SKOR, potassium, cesium, transporter

## Abstract

Potassium (K^+^) is an essential macronutrient for plant growth. The transcriptional regulation of K^+^ transporter genes is one of the key mechanisms by which plants respond to K^+^ deficiency. Among the *HAK/KUP/KT* transporter family, HAK5, a high-affinity K^+^ transporter, is essential for root K^+^ uptake under low external K^+^ conditions. *HAK5* expression in the root is highly induced by low external K^+^ concentration. While the molecular mechanisms of *HAK5* regulation have been extensively studied, it remains unclear how plants sense and coordinates K^+^ uptake and translocation in response to changing environmental conditions. Using *skor* mutants, which have a defect in root-to-shoot K^+^ translocation, we have been able to determine how the internal K^+^ status affects the expression of *HAK5*. In *skor* mutant roots, under K^+^ deficiency, *HAK5* expression was lower than in wild-type although the K^+^ concentration in roots was not significantly different. These results reveal that *HAK5* is not only regulated by external K^+^ conditions but it is also regulated by internal K^+^ levels, which is in agreement with recent findings. Additionally, HAK5 plays a major role in the uptake of Cs^+^ in roots. Therefore, studying Cs^+^ in roots and having more detailed information about its uptake and translocation in the plant would be valuable. Radioactive tracing experiments revealed not only a reduction in the uptake of ^137^Cs^+^ and ^42^K^+^in *skor* mutants compared to wild-type but also a different distribution of ^137^Cs^+^ and ^42^K^+^ in tissues. In order to gain insight into the translocation, accumulation, and repartitioning of both K^+^ and Cs^+^ in plants, long-term treatment and split root experiments were conducted with the stable isotopes ^133^Cs^+^ and ^85^Rb^+^. Finally, our findings show that the K^+^ distribution in plant tissues regulates root uptake of K^+^ and Cs^+^ similarly, depending on *HAK5*; however, the translocation and accumulation of the two elements are different.

## 1 Introduction

To maximize growth in the environment, plants coordinate mineral uptake from the soil. Plant roots have transporters on their cell membranes that are regulated at both transcriptional and post-transcriptional levels. It is necessary to understand molecular mechanisms in order to control ion transport in plants so that they can be used for agricultural purposes such as fertilization and phytoremediation. In plants, potassium (K^+^) is among the most abundant macronutrients corresponding to between 2-6% of their dry mass (Leigh and Wyn Jones, 1984), and it plays a key role in the regulation of osmotic pressure, cytoplasmic pH, membrane potential, and metabolism catalytic activity (Ragel et al., 2019). In plant cells, the K^+^ concentration in the cytoplasm is usually maintained close to 100 mM (Walker et al., 1996). However, the concentration of K^+^ in the soil is highly fluctuating, ranging from micro-millimolar to millimolar (Rengel and Marschner, 2005; Maathuis, 2009). The uptake and distribution of K^+^ in plants are determined by a variety of K^+^ transport systems, which include channels and transporters with varying K^+^ affinity and localization. The K^+^ channels and transporters are composed of multiple gene families that function at different external K^+^ concentrations (Véry and Sentenac, 2003; Véry et al., 2014). In particular, the HAK5 high-affinity transporter is responsible for K^+^ uptake when external K^+^ is above 10 µM, whereas AKT1 is active at more than 100µM. In addition, non-selective cation channels are active at an external K^+^ concentration of more than 1 mM (Gierth et al., 2005; Pyo et al., 2010; Nieves-Cordones et al., 2014). *HAK5* transcript levels rise rapidly during K^+^ deficiency in order to enhance high-affinity K^+^ uptake but decrease during K^+^ sufficiency (Ahn et al., 2004; Gierth et al., 2005). In addition, under low K^+^ conditions, root growth is impaired in HAK5 knockout mutants compared to wild-type (Qi et al., 2008). Also, HAK5, which belongs to the KT/KUP/HAK family, is a major component in mediating high-affinitty K^+^ uptake in Arabidopsis under low K^+^ conditions.

In addition, HAK5 is the main contributor to cesium (Cs^+^) uptake by plants (Qi et al., 2008). Then, the *hak5-1* mutant strongly reduces Cs^+^ uptake and is more tolerant of Cs^+^. In contrast, the *akt1-1* mutant is more sensitive to Cs^+^ than the wild-type due to an increased expression of *HAK5* (Qi et al., 2008). Cs^+^ itself is not required for plant growth. However, the chemical properties of Cs^+^ are similar to those of K^+^, so it is taken up by the plant and perturbs cellular activity (Hampton et al., 2004; Adams et al., 2013). Due to the radioactive Cs^+^ (^134^Cs^+^ and ^137^Cs^+^) spreading from the explosion of the Fukushima Daiichi nuclear power plant, understanding the regulation of HAK5 activity is crucial to producing safe crops by reducing the entry of Cs^+^ into the food chain and utilizing phytoremediation technologies to remove Cs^+^ from the contaminated soil (Nieves-Cordones et al., 2017; Rai et al., 2017).

In the past decade, multiple regulation mechanisms of HAK5 transporter have been identified. Low environmental K^+^ conditions stimulate signaling and enhance the accumulation of transcripts and activity of HAK5. In addition, low external K^+^ concentrations induce plasma membrane hyperpolarization (Nieves-Cordones et al., 2008; Rubio et al., 2014) and extracellular acidification. In turn, extracellular acidification accelerates the H^+^-coupled transport of HAK5. Furthermore, under the low K^+^ conditions, ethylene increased and positively regulated reactive oxygen species (ROS), which in turn induced the transcription factor RAP2.11 gene expression, thereby positively regulating *HAK5* (Shin et al., 2005; Kim et al., 2012). In a recent study, Hong et al. (2013) reported the identification of several transcription factors that could promote *HAK5* expression. It has also been shown that the transcription factor, Auxin Response Factor 2 (ARF2) directly binds to the *HAK5* promoter and repressed *HAK5* expression under K^+^ sufficient conditions (Zhao et al., 2016). In response to low-K^+^ treatment, the DNA-binding activity of ARF2 to the *HAK5* promoter is abolished, promoting *HAK5* transcription (Zhao et al., 2016). HAK5 is also subjected to post-transcriptional regulation. Membrane hyperpolarization and ROS activate Ca^2+^ permeable channels, and this Ca^2+^ signal can be perceived and transduced downstream by a Ca^2+^ sensor such as CBL. CBL binds to CIPK23, a cytoplasmic kinase, which phosphorylates HAK5. The phosphorylation of HAK5 increases its affinity for K^+^ (Ragel et al., 2015).

In order to increase ion uptake and accumulation in plants, the overexpression of ion transporters has been promoted. Nevertheless, it does not always result in higher uptake and biomass (Ai et al., 2009). Therefore, further research is needed to unravel complex regulation mechanisms. At a whole plant scale, the phloem K^+^ concentration provides information about shoot K^+^ demand, and potassium-release channels of the xylem parenchyma take advantage of this signal to coordinate K^+^ uptake (Wegner and De Boer, 1997; Dreyer et al., 2017). In addition, it has been shown that the K^+^ translocation rate affects K^+^ uptake (Nieves-Cordones et al., 2019). It was therefore observed that *skor* knock-out mutants, which are defective in the highly selective outward-rectifying K^+^ channels responsible for releasing K^+^ into xylem sap toward the shoot prevented the uptake of Rb^+^ by plants under K^+^ deficiency conditions (Nieves-Cordones et al., 2019).

In previous studies, it has been found that low K^+^ environmental conditions are associated with a signaling network that is responsible for the regulation of HAK5 at the cellular level (Nieves-Cordones et al., 2008; Rubio et al., 2014; Ragel et al., 2015; Zhao et al., 2016). It is still necessary, however, to conduct further studies in order to be able to gain a better understanding of how K^+^ is sensed. As a whole plant, the roots are the main organs that are directly exposed to the external environment. The low K^+^ signal is first perceived at the plasma membrane of the root epidermal cells and then it is transduced into the cytoplasm of the cells. A short-term response occurs within a few hours without a noticeable change in the cytoplasmic K^+^ level, and a long-term response is stimulated by a decrease in the cytoplasmic K^+^ level. Only the deprivation of K^+^ produces functional HAK5-mediated K^+^ uptake in the root (Rubio et al., 2014). However, there is little information available on whether or not changes in internal K^+^ distribution and/or concentration affect the expression of transporters in roots. In Arabidopsis, SKOR (Stelar K^+^ Outward Rectifier) is expressed in root stele cells (pericycle and xylem parenchyma cells), where it is involved in mediating K^+^ secretion by the xylem parenchyma cells of roots and toward the xylem vessels (Gaymard et al., 1998). SKOR, being an outward-rectifying channel, opens upon membrane depolarization to allow cytosolic K^+^ to be released from the cell. Thus, SKOR plays a significant role in the transport of K^+^ over long distances, especially in the translocation of K^+^ from roots to shoots. Consequently, both *skor* knockout mutants prevented K^+^ from being transported from the root to the shoot, and the shoot K^+^ concentration decreased drastically by 50% compared to the wild-type genotype (Gaymard et al., 1998). In addition, NRT1.5/NPF7 has been described as a proton-coupled H^+^/K^+^ antiporter involved in the translocation of K^+^ from roots to shoots (Li et al., 2017). It should be noted, however, that NRT1.5/NPF7 is able to function under low ${\text{NO}}_{3}^{-}$ availability regardless of the availability of K^+^, whereas SKOR is able to mediate K^+^ translocation from root to shoot when there is low K^+^ availability and high ${\text{NO}}_{3}^{-}$ (Drechsler et al., 2015; Meng et al., 2016; Li et al., 2017).

In this study, we have decided to focus on how K^+^ translocation from roots to shoots impacts the K^+^ and Cs^+^ uptake mechanisms and distribution in plants in the short-term and the long-term responses. The present study demonstrated, using *skor* mutants, that the balance in the distribution of K^+^ between shoots and roots affects the expression of the high-affinity potassium transporter *HAK5* gene in the roots. Furthermore, since HAK5 plays a crucial role in the uptake of Cs^+^, we have evaluated the transport properties of Cs^+^. Interestingly, using these experiments, the ion uptake capacity of AKT1 could be separated from that of HAK5 since AKT1 is not permeable to Cs^+^, therefore inhibiting its activity. (Schachtman, 2000; Adams et al., 2019). Finally, our study showed that SKOR mutation altered the distribution of K^+^ and Cs^+^ in the plant differently.

## 2 Materials and methods

### 2.1 Plant materials and growth conditions

Arabidopsis (*Arabidopsis thaliana*) ecotype Colombia (Col) and Wassilewskija (WS) were used in this study. *skor 1-1* in WS ecotype were kindly provided by Dr. Anne-Alienor Very (INRAE, Montpellier). *skor3-1* (GK391G12) in Col ecotype was obtained from the NASC (Nottingham Arabidopsis Stock Centre). *hak5-1* (SALK_014177) in Col ecotype was obtained from the ABRC (Arabidopsis Biological Resource Centre). T-DNA insertion and homozygous lines were identified by PCR using primers T-DNA left border primer and a gene-specific primer (**Supplementary Table 1**).

For root phenotype assay, seeds were surface sterilized and sown *in vitro* on a Petri dish plate with a solid medium containing low-K^+^ (KCl 10 µM) and high-K^+^ (KCl 1000 µM) for 7 days. Solid medium is composed of nutrient solution (0.75 mM MgSO_4_, 2 mM Ca(NO_3_)_2_, 0.5 mM H_3_PO_4_, 9.25 µM H_3_BO_3_, 3.6 µM MnSO_4_, 3 µM ZnSO_4_, 0.785 µM CuSO_4_, 0.074 µM NH_4_Mo_7_O_24_, 3.5 mM MES, pH 5.8), 0.5% (w/v) Suc, and 0.8% (w/v) agar). For phenotype assay with Cs^+^, seeds were sown *in vitro* on a Petri plate with a solid medium containing low-K^+^ (KCl 10 µM) nutrient solution. Two days after germination, seedlings were transferred to a liquid medium KCl 10 µM with CsCl 0 or 100 µM for 4 days. For the radiotracer experiment, plants were grown for 14 days in solid medium low-K^+^ (KCl 10 µM) and high-K^+^ (KCl 1000 µM) containing nutrient solution. For measurements of K^+^ distribution phenotype between lines and qRT-PCR experiments, plants were grown in a solid medium containing 100 µM K^+^ and nutrient solution mix for 12 days, then transferred to sand culture with nutrient solution containing 100 µM K^+^ for 7 days, then transferred to hydroponic cultures with nutrient solution containing 0, 10, 100, and 1000 µM K^+^ for 4 days. For cold Cs^+^ and Rb^+^ trace experiments, plants were grown in a solid medium containing 100 µM K^+^ and nutrient solution for 12 days, then transferred to sand culture with nutrient solution containing 100 µM K^+^ for 10 days, then transferred to hydroponic cultures using a nutrient solution containing low-K^+^ (KCl 10 µM) and high-K^+^ (KCl 3000 µM) for 8 days, and then was transferred to a nutrient solution containing 10 µM K^+^ (12 plants per 15 L nutrient solution containing 10 µM K^+^) with 1 µM CsCl and 1 µM RbCl for 3 days. Plants were grown in a growth chamber set to 23°C and 8 h Light/16 h dark cycle.

### 2.2 Root morphology analyses

Pictures of the plates were taken with a camera to analyze lateral root density and root lengths. Root length was measured using the plugin NeuronJ (Meijering et al., 2004) for the ImageJ software.

### 2.3 ICP analysis

The roots of plants were washed in sterile distilled water, separated into shoots and roots, and dried for 3 days at 50°C. Dried samples were digested in HNO_3_ (concentrated) at 80°C overnight. After filtration, acid solutions were diluted with 1% HNO_3_. K^+^ concentrations in the solution were determined by ICP-OES (Agilent Technology 5800). Cs^+^ and Rb^+^ were determined by ICP-MS (OPTIMA 8300, Perkin Elmer).

### 2.4 RNA extraction and RT-qPCR

Total RNA was extracted from the shoot and root of 23-day-old plantlets, using Direct-zol RNA MiniPrep (ZYMO RESEARCH). cDNA was synthesized with the qScript cDNA SuperMix (Quanta). Quantitative real-time PCR was conducted with the Light Cycler 480 SYBR Green I Master PCR Mater (Roche) on a Light Cycler 480 (Roche) following the manufacturer’s protocols. The amplification reactions were performed in a total volume of 5 µl, which contained 2 µl cDNA, 2.5 µl SYBR Green premix, and 0.5 µl forward and reverse primers (1µM). The PCR was programmed as follows: 90^°^C for 10 min, followed by 40 cycles of 95°C for 15 s and 60°C for 1 min. For each pair of primers, the PCR efficiency was around 100% and a threshold value was determined. The specificity of PCR amplification was examined by monitoring the presence of the single peak in the melting curves after RT–qPCRs. The relative expression of the gene in each sample was compared to the control sample and was calculated with the delta delta Ct (Ct) method using the following equation: relative expression = 2^–ΔΔCt^, with ΔCt = Ct_sample_ – Ct_control_ and with Ct = Ct_target gene_ – Ct_housekeeping gene_, where Ct refers to the threshold cycle determined for each gene in the exponential phase of PCR amplification (Livak and Schmittgen, 2001). Using this analysis method, the relative expression of the gene in the control sample was equal to one, and the relative expression of the other treatments was then compared to the control plants. The housekeeping gene was the *ROC3* gene (At2g16600) (Bonnot et al., 2016; Genies et al., 2021). The subsequent RT-qPCRs were performed in triplicate for each sample. Primer sequences are provided in **Supplementary Table S1**.

### 2.5 ^137^Cs and ^42^K Uptake measurements

Plants 14 days old grown in solid medium-low-K^+^ (KCl 10 µM) or high-K^+^ (KCl 1000 µM) containing nutrient solution were incubated for 2 hours in hydroponic cultures using a nutrient solution containing 1 µM ^39^K^+^, 1 µM ^133^Cs, and 100 Bq/ml ^137^Cs or a mixed tracer of ^42^K and ^43^K 500 Bq/ml. At the end of the uptake period, plant roots were washed with 1 mM KCl and 1 mM CsCl solution. The radioactivity in plants was measured with a gamma counter (AccuFLEXγ8000, HITACHI.co). For the gamma-ray measurements of the ^42^K (half-life,12 hours) and ^43^K (half-life, 22 hours) RI mixture, only the spectrum of ^43^K was measured during the experiments due to its longer half-life.

### 2.6 Split root experiment

Plants were grown under the same conditions as those used for the cold trace experiments of Cs^+^ and Rb^+^. Then, the roots were washed in distilled water and separated into two parts. The two root parts were maintained in the same nutrient solution mix containing low -K^+^ (KCl 10 µM) for 8 days. The tracer experiment was conducted by placing two root parts in separate pots. Then, 1 µM CsCl or 1 µM RbCl was added to one part of the root for 3 days. Mineralization and measurement are the same as the method described in the ICP analysis.

### 2.7 Accession numbers

Sequence data for the genes described in this article are in the Arabidopsis TAIR database (https://www.arabidopsis.org/index.jsp) under the following accession numbers: At3g02850 for *SKOR*, At4g13420 for *HAK5*, At2g30070 for *AtKUP1*, At2g40540 for *AtKUP2*, At3g02050 for *AtKUP3*, At4g23640 for *AtKUP*4, At4g33530 for *AtKUP5*, At1g70300 for *AtKUP6*, At5g09400 for *AtKUP7*, At5g14880 for *AtKUP8*, At4g18860 for *AtKUP9*, At1g31120 for *AtKUP1*0, At2g35060 for *AtKUP11*, and At1g60160 for *AtKUP12*.

## 3 Results

### 3.1 The *skor* mutant is tolerant to low K^+^ stress

**Figure 1:**
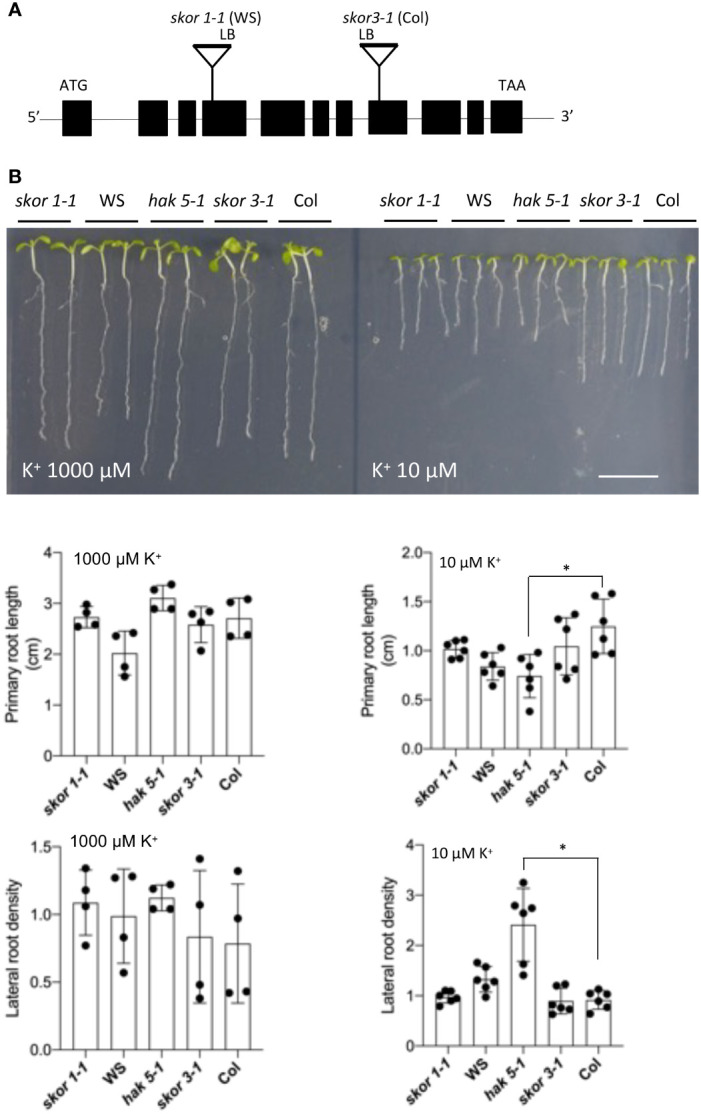
K^+^-dependent growth analysis of *skor* T-DNA insertion mutants. **(A)**. A schematic representation of the T-DNA insertion in the *SKOR* gene (At3g02850.1) in the WS or Col-0 ecotypes. A solid line and black boxes indicate exons and introns, respectively. The T-DNA insertion sites in *SKOR* are represented as triangles, and the left border (LB) orientation is indicated. **(B)**. In contrast to wild-type plants, both *skor-1* and *skor-3* mutants show no phenotype when grown for 7 days on a medium containing KCl at the indicated concentration. The scale bar is 1 cm. The lateral root density and the length of the primary roots were determined for wild-type and skor mutant plants after 7 days. Each bar represents the mean root length (n = 4-6) of seedlings ± SD. Statistical significance was determined by Student’s t-test. Significant differences between Col and *hak5-1* mutants are indicated with asterisks (*P <0.05).

We determined the root growth of *skor* mutants under different K^+^ conditions. This study used two independent knockout mutants of the highly selective outward-rectifying K^+^ channel SKOR including *skor1-1* in the WS background (Gaymard et al., 1998) and *skor3-1* in the Col background. As shown in **Figure 1**, both *skor* mutants showed similar root architecture to the wild type both under high (1000 µM) and low (10µM) K^+^ conditions. In contrast, in *the hak5-1* mutant, primary root growth is strongly impaired and a significant increase in lateral root density is observed under low K^+^ conditions (10 µM) (**Figure 1**), as previously observed (Qi et al., 2008). These results show that the root morphology of the *skor* mutants is not modified by low K^+^ conditions compared to *hak5-1*.

### 3.2 *HAK5* transcription in *skor* mutants decreased in low K^+^ conditions

**Figure 2:**
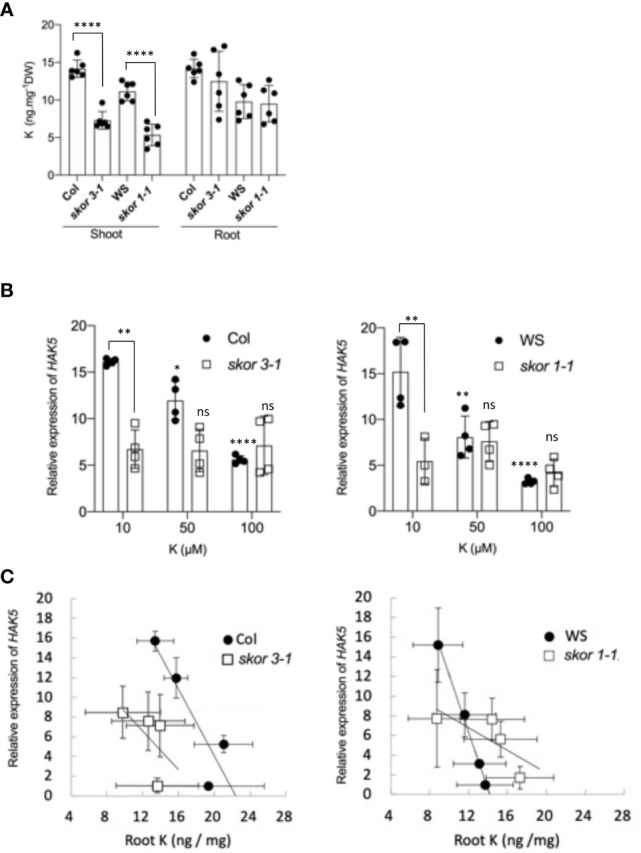
Both the external and internal concentrations of K^+^ affect the expression of *HAK5* in roots. **(A)**. SKOR mediates K^+^ translocation to the shoot. The K^+^ concentration of roots and shoots of different genotypes was measured using ICP-OES. Wild-type and mutant plants were grown in hydroponic cultures for 5 weeks in a medium containing 0.1 mM KCl. Data represent the mean ± SD (n = 6). Statistical significance was determined by Student’s t-test with Welch’s correction, and significant differences are indicated with asterisks (****P < 0.0001). **(B)**. RT-qPCR analyses of *HAK5* expression in roots of wild-type and *skor* mutants. *HAK5* expression was negatively correlated with external potassium concentration in the roots of both wild-type ecotypes. Asterisks represent statistical significance between K+ treatments based on two-way ANOVA  analysis (*p = 0.0227, **p = 0.0012, ****p < 0.0001) and statistical significance between genotypes bases on t-test with Welch's correction (**p < 0.01). **(C)**. A comparison of the expression levels of *HAK5* in WT and the *skor* mutant according to the K^+^ concentration in roots. The correlation analysis was performed, and Pearson’s Correlation Coefficient square using z scores was also calculated using Excel Software (Microsoft) electronic datasheet (Pearson’s Correlation Coefficient: Col r = - 0,57, p-value = 0,0005; *skor3-1*: r = - 0,34, p-value = 0,4095; WS: r = - 0,51, p-value = 0,0023; *skor1-1*: r = - 0,11, p-value = 0,7663). The expression of *HAK5* is negatively correlated with the root’s internal potassium concentration in wild-type plants, whereas these correlations are lost in *skor* mutants.

To determine and compare the transcriptional regulation of the KUP/KT/HAK transporters in response to K^+^ concentration, plants were grown under different K^+^ conditions, ranging from 10 µM to 1000 µM. Among all members of the family, only *HAK5* is highly induced in roots when the external K^+^ concentration is less than 100µM, and no expression is detected above this concentration (**Supplementary Data 1**, **2**). Previous studies have reported similar results (Ahn et al., 2004, Shin and Schachtman, 2004, Gierth et al., 2005). In contrast, transcription of the *SKOR* gene, which was predominantly detected in roots, did not change in roots according to K^+^ conditions (**Supplementary Data 2**). However, under low (10µM) K^+^ environmental conditions, the expression level of *HAK5* in roots is strongly decreased in *skor* mutants compared to the wild types (**Figure 2B**). These results suggest that a decrease in K^+^ concentrations in the shoots of *skor* mutant plants (**Figure 2A**) induces a modification of the potassium distribution between roots and shoots, increasing the root-to-shoot ratio. This increase might affect the *HAK5* expression in roots. In order to determine whether the decrease in *HAK5* transcription levels in *skor* mutants is associated with an increase in K^+^ concentration in roots, the K^+^ concentrations in the roots of plants exposed to various K^+^ concentrations were measured using ICP-OES, and the correlation between K^+^ concentrations and *HAK5* transcription levels was calculated. Then, correlation analysis between the relative expression of *HAK5* and the root K^+^ concentrations (**Figure 2C**) was studied and the Pearson’s Correlation Coefficient using z scores was determined using Excel Software (Microsoft) electronic datasheet. The results show a correlation between *HAK5* expression and K^+^ concentrations in wild-type Col roots (r = - 0,57, p-value = 0,0005) and the wild-type WS roots (r = - 0,51, p-value = 0,0023). This linear regression, however, is lost in both *skor* mutants (**Figure 2C**), *skor 3-1* (r = - 0,34, p-value = 0,4095) and *skor1-1* (r = - 0,11, p-value = 0,7663). Overall, these results suggest that in addition to external K^+^ conditions, K^+^ root concentrations tightly regulate *HAK5* expression in roots.

### 3.3 *skor* and *hak5* mutants are more Cs^+^ tolerant

**Figure 3:**
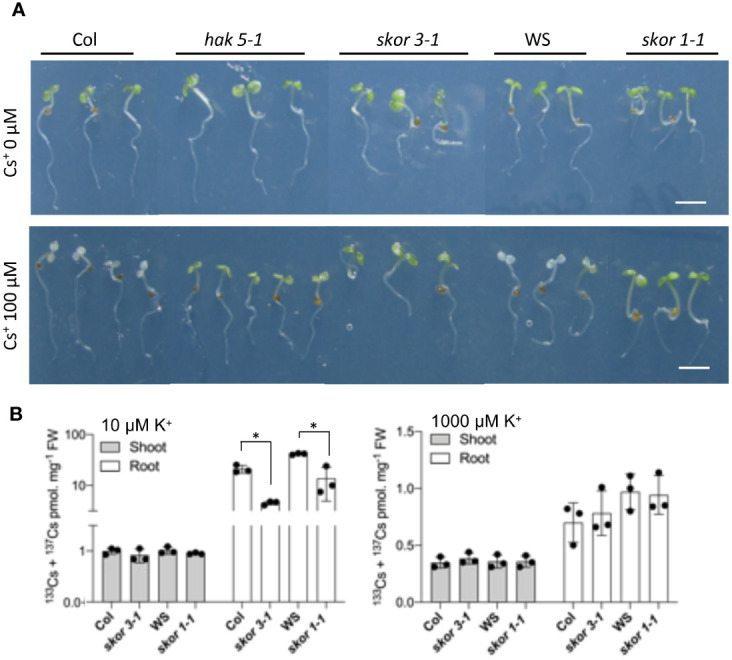
*skor* mutants are tolerant to Cs^+^ due to a decrease in Cs^+^ uptake. **(A)**. Wild-type (Col, WS), both *skor* mutants *skor3-1* (Col), *skor1-1* (WS), and *hak5-1* were germinated for 4 days and then transferred into a hydroponic medium containing different Cs^+^ concentrations for 4 days. The control plants showed chlorosis as a result of Cs^+^ toxicity, whereas the *skor* and *hak5-1* mutants were more resistant. The scale bar was 0.5 cm. **(B)**. Plants were grown on an agar medium containing different K^+^ concentrations for 14 days. Then, roots were incubated for 2 hours in a hydroponic medium containing K^+^ and ^133^Cs 1 μM + ^137^Cs 100 Bq/ml. Cs^+^ uptake is reduced in both *skor* mutants under low K^+^. Data are shown as means ± SD (n = 3). Statistical significance was determined by Student’s t-test with Welch’s correction. Significant differences between wild-types and *skor* mutants are indicated with asterisks (*P < 0.05).

As HAK5 is the major contributor to Cs^+^ uptake in Arabidopsis, we examined Cs^+^ tolerance in both *skor* and *hak5* mutants and their corresponding wild types. Seedlings of wild-type (WS and Col), *skor*, and *hak5-1* knockout mutants were grown in hydroponic cultures for 4 days in the presence of CsCl (100 µM). The *skor* and *hak5* mutants were more resistant to Cs^+^ toxicity than wild-type plants, as shown in **Figure 3A**. In both wild-type ecotypes, Col and WS, the cotyledons bleached significantly in the presence of 100 µM Cs^+^, whereas the cotyledons of *skor1-1*, *skor3-1*, and *hak5-1* remained green. As expected, and previously observed, the *hak5-1* mutant is less susceptible to Cs^+^ toxicity due to its reduced uptake of Cs^+^ (Qi et al., 2008). Furthermore, both *skor* mutants exhibit enhanced resistance to Cs^+^ toxicity, similar to *hak5-1*. Our results were confirmed by measuring the Cs^+^ concentration of plants previously grown under different K^+^ conditions for 14 days. The roots of previously cultivated plants under different K^+^ conditions were exposed for 2 hours in the presence of ^137^Cs in order to determine the uptake rates of each mutant. **Figure 3B** shows that *skor1-1* and *skor3-1* mutant roots showed substantially and significantly reduced Cs^+^ concentration under low K^+^ conditions (10 µM K^+^). However, under high potassium conditions, there was no difference between wild-type and *skor* mutants in Cs^+^ uptake (**Figure 3B**).

### 3.4 The uptake and translocation of K^+^ and Cs^+^ are different

**Figure 4:**
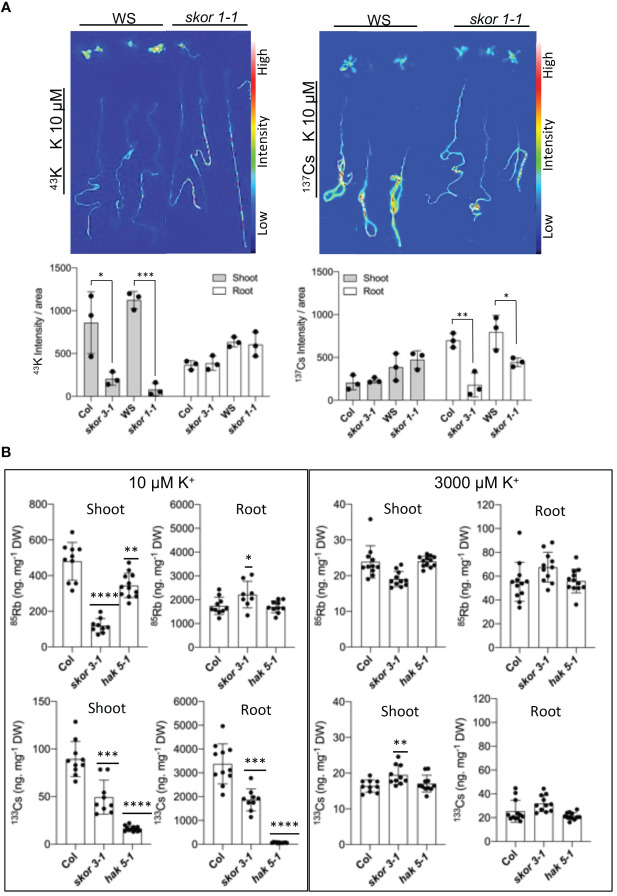
SKOR mutation affects K^+^ and Cs^+^ distribution in plants. **(A)**. Differences in the distribution of K^+^ and Cs^+^ as determined by ^137^Cs and ^42^K. The roots of 12-day-old plants grown in low-K conditions (10 µM) were incubated for 2 hours in a hydroponic medium that contained ^42^K, ^43^K, or ^137^Cs. Data are shown as means ± SD (n = 3). Statistical significance was determined by Student’s t-test with Welch’s correction. Significant differences are between wild types and *skor* mutants indicated with asterisks (*P <0.05). **(B)**. Plants 35 days old were transferred to different K^+^ conditions for 3 days with 1 µM CsCl and 1 µM RbCl in a hydroponic medium. By using ICP-MS, the concentrations of Cs^+^ and Rb^+^ were determined. A decrease in Rb^+^ concentration is observed in the shoots of *skor*. In addition, Cs^+^ concentration is reduced in both shoots and roots of the *skor* mutant. Data are shown as means ± SD (n = 9-12). Statistical significance was determined by Student’s t-test with Welch’s correction. Significant differences between wild types and mutants are indicated with asterisks (*P <0.05, **P = 0.0016, ***P = 0.0002, and ****P < 0.0001).

By mediating K^+^ secretion by the xylem parenchyma cells of roots and toward the xylem vessels, SKOR plays a role in root-to-shoot K^+^ translocation. In consequence, both *skor* knockout mutants significantly decreased K^+^ translocation from roots to shoots, resulting in a substantial decrease of more than 50% in shoot K^+^ concentration (**Figure 2A**). To determine whether SKOR affects Cs^+^ transport in a similar manner, we conducted experiments using radioactive tracers, ^42^K^+^ and ^137^Cs^+^, on plants grown under low potassium conditions to compare K^+^ and Cs^+^ uptake properties. In the *skor1-1* shoot, ^42^K^+^ distributions are significantly impaired compared to the wild type (**Figure 4A**). The distribution pattern of ^137^Cs^+^ between shoots and roots, however, is different from that of ^42^K^+^, with a strong accumulation occurring in the roots of wild-type plants. This high accumulation of ^137^Cs^+^ in the roots is impaired in the *skor1-1* mutant (**Figure 4A**). Interestingly, ^137^Cs^+^ translocation from roots to shoots is higher compared to ^42^K^+^ in *skor* mutants (**Figure 4A**). The shoot:root ratio of K^+^ is higher than that of Cs+ in the wild type, while the shoot:root ratio of *skor* is higher in Cs^+^ than in K^+^ (**Table 1**). According to these findings, we show that in *skor* mutants, Cs^+^ uptake in roots is dramatically reduced, while its translocation to the shoot is not affected. Thus, it is possible that under low K^+^ conditions, the decrease in *HAK5* expression in the *skor1-1* mutant could negatively affect Cs^+^ uptake; however, other factors are also involved in Cs^+^ translocation from roots to shoots. In order to better understand K^+^ and Cs^+^ translocation, accumulation, and repartitioning in plants, long-term treatments (3 days) were performed using ^133^Cs^+^ and ^85^Rb^+^ since ^42^K^+^ tracer has a short half-life of 12.4 hours and so cannot be used for long-term experiments. The roots and shoots of 35-day-old plants were collected separately after 3 days of treatment with ^133^Cs^+^ and ^85^Rb^+^. Plants grown in high K^+^ accumulate significantly less ^133^Cs^+^ and ^85^Rb^+^ than plants grown in low K^+^ (**Figure 4B**). Compared to wild-type shoots, the ^85^Rb^+^ xonxen in the *hak5-1* mutant decreased under low K^+^ conditions, but this was not observed in roots. In the shoots in the *skor3-1* mutant, the ^85^Rb^+^ concentration drastically decreases, as we observed previously, and increases slightly in the root (**Figure 4B**). These results confirm that the translocation of ^85^Rb^+^ from roots to shoots is reduced in the *skor* mutant compared to the wild type. However, this increase in root concentration was not observed in the ^42^K potassium uptake experiment (**Figure 4A**). Furthermore, the ^85^Rb^+^ translocation from roots to shoots is higher in the *hak5-1* mutant than in the *skor* mutant. These results suggest that although ^85^Rb^+^ may be used as a tracer for potassium, the two elements may be transported differently. In contrast, the ^133^Cs^+^ concentration of roots and shoots of the *hak5-1* mutant was significantly reduced. In the *skor3-1* knockout mutant, the ^133^Cs^+^ concentration of roots and shoots was also significantly decreased, but the changes are less pronounced. It is interesting to note that in older plants of the *skor* mutants, not only the translocation of ^133^Cs^+^ from roots to shoots is reduced as observed in **Figure 4A** but also its concentration in the roots (**Figure 4B**, **Table 2**). Altogether, these results suggest that under low K^+^ conditions, root uptake of K^+^ and Cs^+^ is similar and may be affected by *HAK5* expression; however, their translocation and their accumulation are different.

**Table 1:** Shoot/Root ratios of ^43^K^+^ and ^137^Cs^+^.

	^43^K^+^	^137^Cs^+^
Col	2.44 ± 1.30	0.30 ± 0.16
*skor 3-1*	0.52 ± 0.08	2.04 ± 1.61
WS	1.77 ± 0.07	0.47 ± 0.09
*skor 1-1*	0.14 ± 0.12 ^***^	1.08 ± 0.30 ^*^

Root:shoot ratios of ^43^K^+^ and ^137^Cs of WT and *skor* plants calculated from **Figure 4A**. Treatments are described in **Figure 4**. Data are shown as means ± SD (n=3). Statistical significance was determined by Student’s t-test with Welch’s correction. Significant differences between wild-types and skor mutants are indicated with asterisks (*P <0.05, ***P=0.0002).

**Table 2:** Shoot/Root ratios of ^85^Rb^+^ and ^133^Cs^+^.

	^85^Rb^+^		^133^Cs^+^	
	K^+^ 10	K^+^ 3000	K^+^ 10	K^+^ 3000
Col	0.28 ± 0.06	0.46 ± 0.13	0.03 ± 0.01	0.70 ± 0.21
*skor 3-1*	0.06 ± 0.02 ^****^	0.28 ± 0.09 ^**^	0.03 ± 0.01	0.63 ± 0.20
*hak 5-1*	0.20 ± 0.04 ^**^	0.44 ± 0.11	0.23 ± 0.08 ^****^	0.81 ± 0.15

Root:shoot ratios of ^85^Rb^+^ and ^133^Cs of Col, *skor3-1* and *hak5-1* plants calculated from **Figure 4B**. Treatments are described in **Figure 4B**. Data are shown as means ± SD (n=9-12). Statistical significance was determined by Student’s t-test with Welch’s correction. Significant differences between wild-types and mutants are indicated with asterisks (**P < 0.005, ****P < 0.001).

### 3.5 Ion circulation keeps K^+^ status higher in the root in the *skor* mutant.

**Figure 5:**
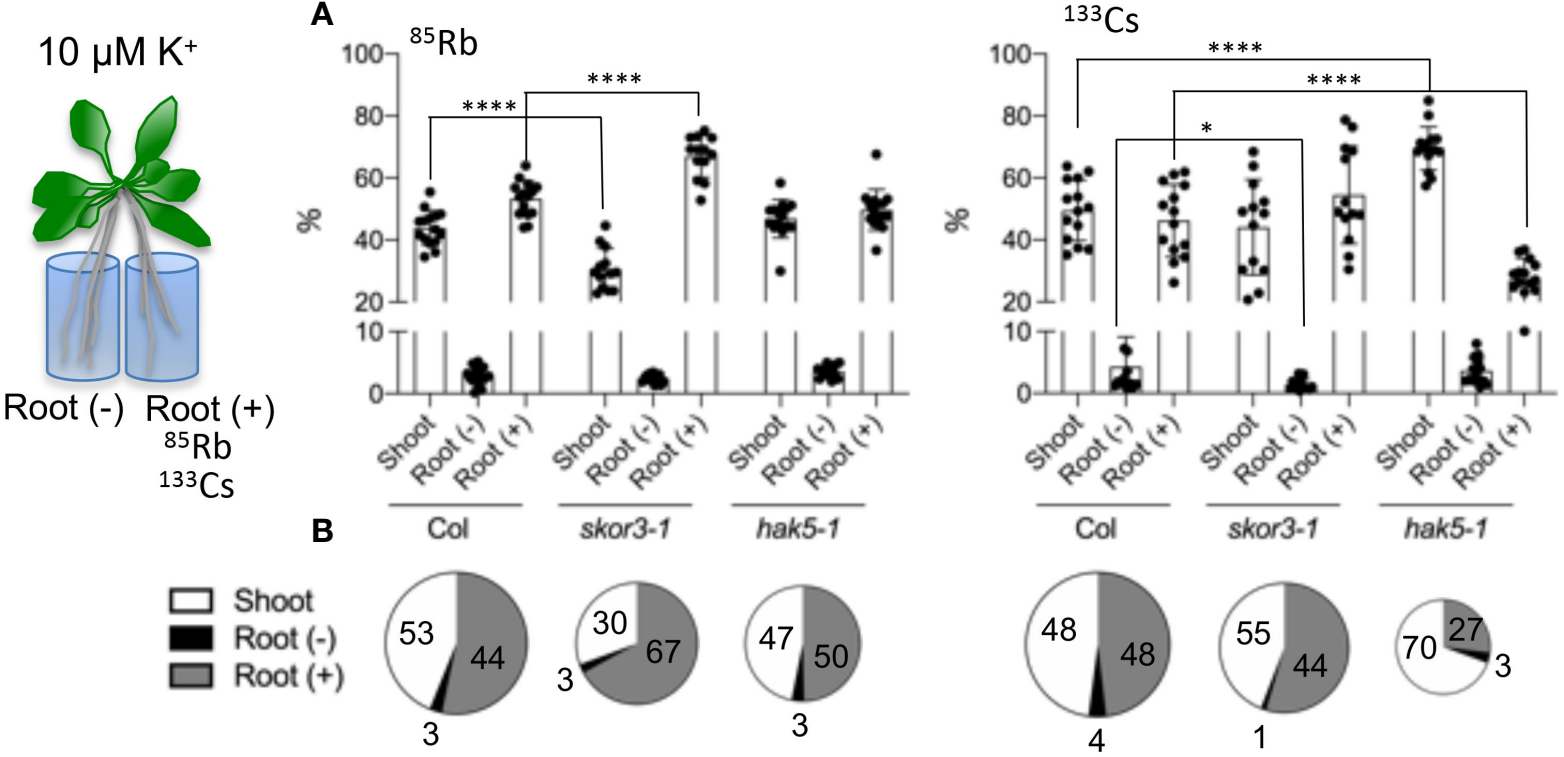
An analysis of the Rb^+^ and Cs^+^ distribution in mutants. A long-term study of ion distribution in plants. *skor* did not exhibit similar effects on the translocation of ^133^Cs and ^85^Rb from root to shoot. Plant roots 35 days old are divided into two parts, and one part is incubated with 1µM ^133^Cs or 1 µM ^85^Rb (Root +) for 3 days. The number of ions in the three parts was determined using ICP-MS. **(A)**. Plant distribution (%) of ^85^Rb and ^133^Cs among the three parts of the plant: shoot, root (+) incubated with 1µM ^133^Cs or 1µM ^85^Rb, and root (-). **(B)**. An illustration of the distribution of the data from A in a pie chart. According to the pie chart, the size corresponds to the total amount of ions absorbed. Data are shown as means ± SD (n=11-15). Statistical significance was determined by Student’s t-test with Welch’s correction. Significant differences between wild types and mutants are indicated with asterisks (*P =0.0457 and ****P <0.0001).

There is a constant movement of potassium through the tissues and organs of plants, which is transported in both directions at the same time, upstream by the xylem and downstream by the phloem. Therefore, split root experiments were performed to determine the impact of K^+^ or Cs^+^ fluxes on the distribution of K^+^ and Cs^+^ between the roots and shoots (**Figure 5**, **Supplementary Data 3**). Split roots were cultivated in the solution containing ^133^Cs^+^ or ^85^Rb^+^ for 3 days, and subsequently collected in three parts: shoots, roots with tracer, and roots without tracer, and percentages were calculated for each part (**Figure 5**). The results reveal that wild-type plants showed similar distributions (%) of applied Rb^+^ and Cs^+^ in shoots and half roots (treated and untreated), indicating that both Cs^+^ and K^+^ circulate within the plant. In the *skor3-1* mutant, there is a slight reduction in the total uptake of Rb^+^ or Cs^+^. In addition, less Rb^+^ was transported to the *skor3-1* shoot, as previously observed, whereas it was similarly transferred to the untreated root as in the wild type. However, Cs^+^ transported to the shoots of *skor3-1* was close to that of the wild type, whereas Cs^+^ transferred from the shoots to the untreated roots was reduced compared to the wild-type plants. In contrast, in the *hak5-1* mutant, despite a slight decrease in the total Rb^+^ absorption, the Cs^+^ uptake has been dramatically reduced. In the presence of Rb^+^, *hak5-*1 plants exhibit broadly comparable distributions (%) to wild-type plants; however, this distribution is significantly altered when Cs^+^ is applied. In line with previous experiments, the percentage of Cs^+^ transported from the treated roots to the shoots was dramatically increased (70%) compared to the wild type (48%), but the percentage transported from the shoots to the untreated roots remained unchanged. In the present study, we found that when plants are exposed to low K^+^ conditions, HAK5 plays a critical role in the uptake and distribution of Cs^+^ and that SKOR participates in the distribution of Cs^+^ from the shoot to the roots.

## 4 Discussion

Proper control of ion uptake is one of the essential elements for plant growth. It is nevertheless important to note that the regulation of ion transport is rather complex, particularly as a result of the redundancy of genes. For example, 71 K^+^ channels and transporters have already been identified in *Arabidopsis thaliana* (Mäser et al., 2001; Véry and Sentenac, 2003; Amtmann et al., 2018; Wang and Wu, 2013) and divided into six distinct gene families consisting of three channel families and three transporter families (Gierth and Mäser, 2007; Chanroj et al., 2012; Gomez-Porras et al., 2012). K^+^ is absorbed in the root by the epidermis and root hairs. In order to reach the shoot, it must pass through several layers of root cells in order to be transported into the xylem. In order to achieve this process, numerous K^+^ channels and transporters must be present in the epidermis and xylem, which ensures that K^+^ can flow from the epidermis to the xylem. AKT1 and HAK5, two high-affinity K^+^ transport proteins, have been characterized (Hirsch et al., 1998; Rubio et al., 2008), and their regulation mechanisms have been extensively investigated (Rubio et al., 2010; Nieves-Cordones et al., 2014; Feng et al., 2021; Huimin et al., 2021). Compared to K^+^ uptake, little has been known about K^+^ transport in the stele since the identification of SKOR (Gaymard et al., 1998) and NRT1.5/NPF7.3 (Li et al., 2017). Nevertheless, NRT1.5/NPF7.3 is thought to maintain root-to-shoot translocation of K^+^ only under limited ${\text{NO}}_{3}^{-}$ availability (Drechsler et al., 2015; Meng et al., 2016; Li et al., 2017). During K^+^ deficiency, HAK5 exhibits crucial roles in K^+^ uptake and transport, while SKOR plays a prominent role in root-to-shoot K^+^ translocation. However, the details of the relationships between K^+^ uptake, translocation, and recycling are not completely understood. Accordingly, we investigated the molecular and physiological responses of Arabidopsis wild-type (Col and WS), *hak5*, and *skor* T-DNA insertion lines to various K^+^, Cs^+^, and Rb^+^ conditions.

### 4.1 HAK5 expression is regulated by internal K^+^ distribution

There is a close relationship between root uptake and xylem K^+^ loading. There is, however, little understanding of the molecular mechanisms involved in the coordination of these two processes. In this study, a correlation was found between the level of *HAK5* expression and the concentration of K^+^ measured in roots. When the concentration of K^+^ in roots decreases, the expression of *HAK5* increases (**Figure 2**). In addition, we observed that *HAK5* was down-regulated in *skor* mutants. In these mutants, the translocation of K^+^ from root to shoot is impaired, resulting in a significant reduction in shoot K^+^ concentration (**Figures 2**, **4**). Altogether, these results suggest the hypothesis that xylem K^+^ loading regulates K^+^ uptake through the regulation of *HAK5* expression. Changes in the expression of *HAK5* have also been observed in other mutants affected by K^+^ translocation including *nrt1.5-5* (Drechsler et al., 2015; Meng et al., 2016) and in *cpr5* mutants (Borghi et al., 2011). This transcriptional regulation provides a regulatory pathway for long-distance K^+^ signaling during low K^+^ stress and demonstrates that uptake and translocation of K^+^ are closely coordinated. It is noteworthy that AKT1 is not involved in this process as its role in K^+^ resorption from xylem vessels has only been observed under conditions in which K^+^ is sufficient (Nieves-Cordones et al., 2019). By combining genetics, radiotracer experiments, and ion concentration measurements, we have established that K^+^ tissue distribution tightly controls *HAK5* expression.

### 4.2 SKOR does not transport Cs^+^ but contributes to Cs^+^ uptake through HAK5

The chemical properties of Cs^+^ are similar to those of K^+^, and early studies indicate that the mechanisms underlying Cs^+^ and K^+^ uptake in plants are similar (Collander, 1941; Epstein and Hagen, 1952). Furthermore, the level of K^+^ supply also impacts the contribution of each pathway to the uptake of Cs^+^. To date, Cs^+^ uptake has been demonstrated for several K^+^ transporters, including HAK5 (Nieves-Cordones et al., 2017; Rai et al., 2017; Rai and Kawabata, 2020). Additionally, SKOR, which mediates K^+^ xylem loading, has been suggested as a possible candidate for proteins that mediate Cs^+^ translocation from root to shoot. The results of our experiments clearly showed that K^+^ and Cs^+^ translocation rates were affected differentially in *skor* mutants in comparison to wild types (**Figure 4**). Electrophysiology studies indicate that the ratio of Cs^+^ to K^+^ permeability of the SKOR channel in Arabidopsis is 0.15 (Gaymard et al., 1998; Johansson et al., 2006). Thus, the Cs^+^ permeability of SKOR is significantly lower than the K^+^ permeability. In this study, we found that ^137^Cs uptake is strongly decreased in roots but not in shoots under low K^+^ conditions in both *skor* mutants. Thus, it is tempting to speculate that this decrease is caused by the down-regulation of *HAK5* expression. Previous studies have demonstrated that Cs^+^ blocks AKT1 activity, so the role of AKT1 in this process can be excluded (Adams et al., 2019).

### 4.3 Difference of Rb^+^ and Cs^+^ movement

**Figure 6:**
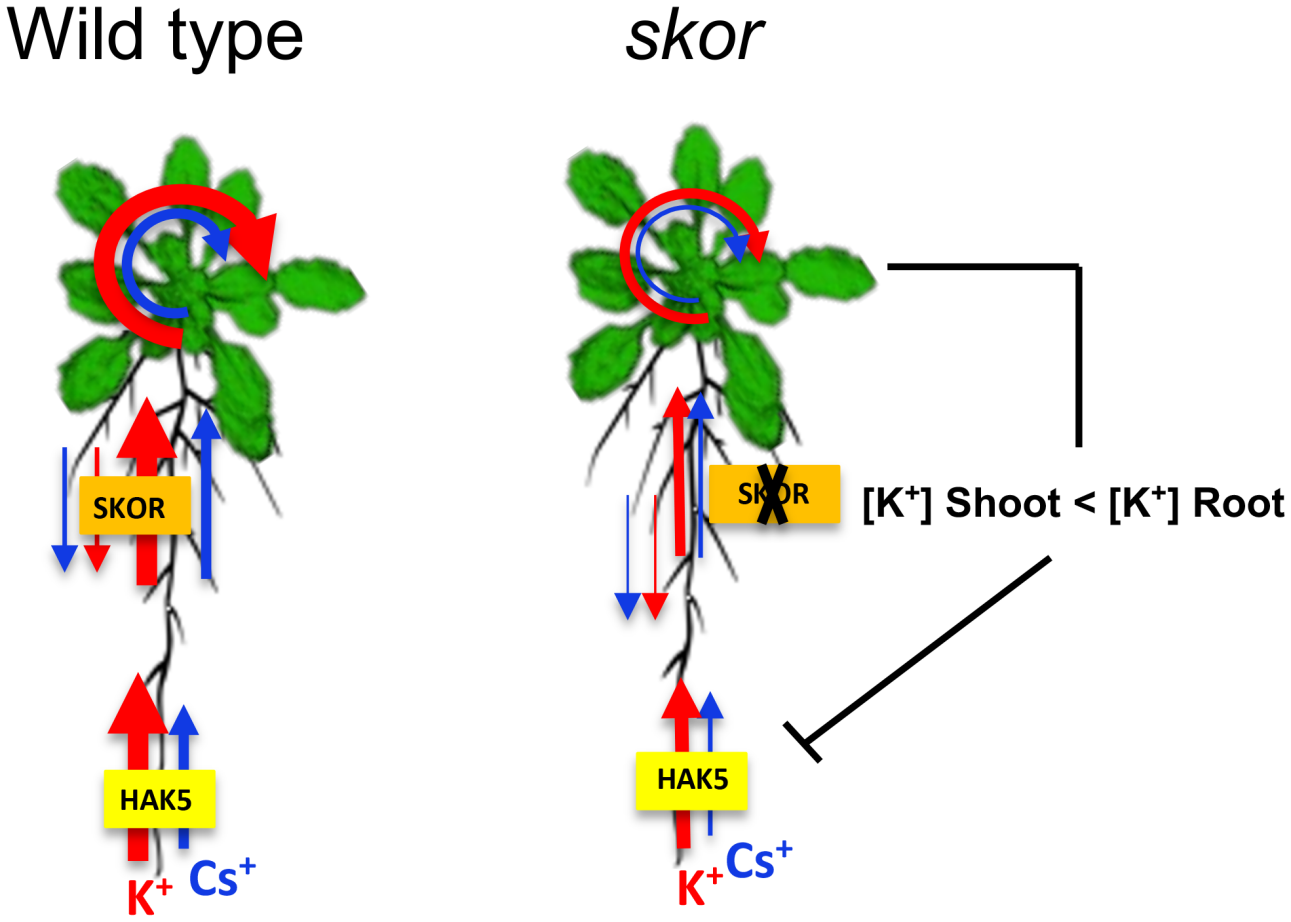
Hypothetical model of the regulation of *HAK5* in *skor* mutants under low K^+^ conditions. This schematic representation illustrates the hypothetical role of SKOR on K^+^ and Cs^+^ uptake and translocation in plants. The size of the arrows represents K^+^ (in red) and Cs^+^ (in blue) fluxes. A lack of the *SKOR* gene reduced the translocation of K^+^ from root to shoot. Since potassium transport from shoot to root is the same in WT and *skor*, the K^+^ in the root is relatively higher in *skor*. As a result of the imbalance in K^+^ concentrations between shoots and roots, *HAK5* was repressed in the roots of *skor* mutants affecting both K^+^ and Cs^+^ absorption. There is, however, a difference in the translocation and distribution of K^+^ and Cs^+^.

A difference in *skor* K^+^ transport was observed in **Figure 4** when comparing results from short-term (2 hours) experiments on young plants and long-term (3 days) experiments on old plants. However, a difference in Cs^+^ accumulation in roots was only observed in old plants. This suggests that Cs^+^ transport to the roots may take longer than K^+^ or that the activity of the transporters may differ with plant age. The analyses of the relative distribution of Rb^+^ and Cs^+^ using split root experiments (**Figure 5**) indicated that the total amount of Rb^+^ decreased similarly in *skor* and *hak5* mutants. In accordance with our radiotracer experiments, both mutants exhibit differential distributions between shoots and roots. Interestingly, there is an increase in the amount of Rb^+^ in the *skor* mutant that coincides with a decrease in *HAK5* expression in the roots. The results of this study confirm the role played by SKOR in regulating K^+^ uptake and the existence of cross-regulation with HAK5 at low K^+^ levels. Alternatively, both mutants exhibit a different pattern of Cs^+^ distribution and amount. In the *hak5* mutant, the Cs^+^ amount was drastically reduced and distributed differently compared to *skor* and Col. A hypothetical model illustrating the regulation mechanism of *HAK5* expression in *skor* is proposed in **Figure 6**. Our results not only show that HAK5 plays a major role in Cs^+^ uptake in roots under low K^+^ conditions but also demonstrate that other transporters are involved in Cs^+^ uptake, translocation, and recycling. Interestingly, two ATP-binding cassette (ABC) proteins, ABCG37 and ABCG33, have been identified as high-affinity Cs^+^ transporters which are K^+^-independent (Ashraf et al., 2021). However, further research is needed to evaluate their contribution to Cs^+^ uptake compared to HAK5 under low K^+^ conditions. Among the candidates likely to be involved in the root-growth translocation of Cs+, the K+ transporter KUP7 is thought to be involved in K^+^ uptake and accumulation in the xylem parenchyma and affects K^+^ loading into the xylem (Han et al., 2016; Šustr et al., 2020). It is known that NRT1.5 is involved in potassium xylem loading, but its discrimination between Cs^+^ and K^+^ is unknown (Drechsler et al., 2015; Li et al., 2017). On the other hand, ZIFL2 (Zinc-induced facilitator-like transporter 2) plays a role in the partitioning of Cs^+^. A mutation in ZIFL2 results in the accumulation of Cs^+^ in shoots (Remy et al., 2015). KT/HAK/KUP transporters have recently been identified to accumulate potassium in rice xylem parenchyma, such as OsHAK5, which contributes to both potassium uptake and xylem loading (Yang et al., 2014). However, this transporter is not involved in Cs accumulation. Furthermore, it cannot be excluded that HAK5 also contributes to the loading of K^+^ and Cs^+^ into the xylem. Finally, we investigated the role of KUP6 in K^+^ translocation for Cs^+^ transport by using a *kup6* T-DNA insertion mutant. Nevertheless, the kup6 single mutant showed no significant difference in Cs^+^ translocation (data not shown).

Finally, we present new insights regarding long-distance K^+^ and Cs^+^ transport, which may contribute to increased fertilizer efficiency and application for phytoremediation. Furthermore, our results indicate that modifying only the surface transporters in the root is not sufficient to improve fertilizer efficiency or facilitate phytoremediation. Further research is required to understand the regulatory mechanism of root surfaces and internal transporters in coordination with each other, which is crucial for the above applications.

## Data availability statement

The datasets presented in this study can be found in online repositories. The names of the repository/repositories and accession number(s) can be found in the article/**Supplementary Material**.

## Author contributions

NL and AV designed the research. NL, SK, LM, NV, SC, TN, and JF performed the research. NL and SK analyzed the data and wrote the paper with the contribution of all authors. All authors contributed to the article and approved the submitted version.
