# Supplementary material for: Xylem K+ loading modulates K+ and Cs+ absorption and distribution in Arabidopsis under K+-limited conditions
Source: Front Plant Sci. 2023 Sep 22;14:1040118. doi: 10.3389/fpls.2023.1040118 (PMC10557132; doi:10.3389/fpls.2023.1040118)
Supplement: Supplementary file 1 [file DataSheet_1.pdf]

Supplemental Data

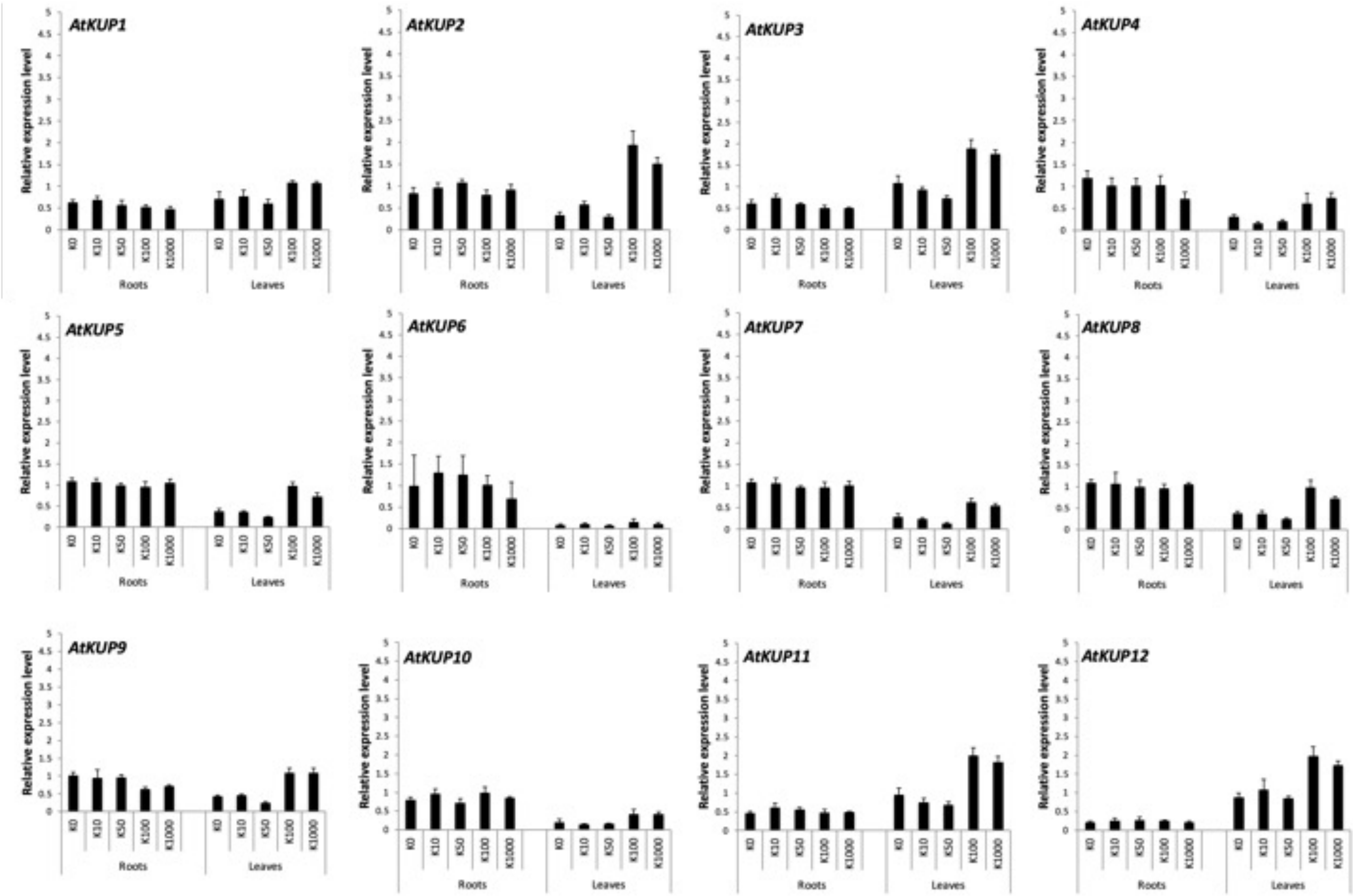

Sup. Figure 1. RT-qPCR analysis of KUP/KT/HAK family transporter expression in Wild type.

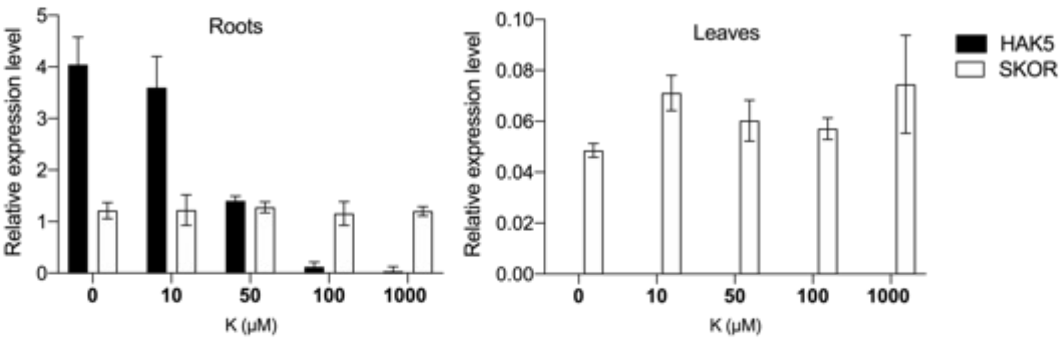

Sup. Figure 2. Comparison between *HAK5* and *SKOR* expression in Wild type.

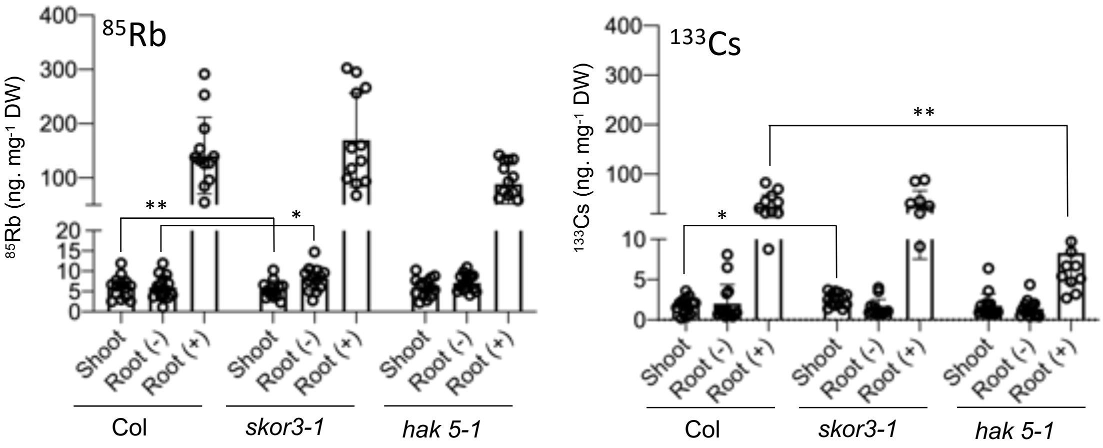

Sup. Figure 3. Rb^+ and Cs^+ distribution between three parts.

Sup.Table1. Primer list

Primer name	Primer sequence
AtHAK5_F	ATCTAATGGGAGAGACCGAG
AtHAK5_R	GTTGGCATGACCTATGAGTT
AtKUP1_F	CGAAAGAAGCTGGAGTAGC
AtKUP1_R	GTTCCTCACACTTCTCTGC
AtKUP2_F	CGGTGATGAAGAGATTAGCAG
AtKUP2_R	GAAGTCGGCATGGTTTATG
AtKUP3_F	CACTCGTATGTGAAATCGAGG
AtKUP3_R	CTCACATCAGCCTCATTGA
AtKUP4_F	CAATGGACACCAAGTTCAGG
AtKUP4_R	GCGTACTCGTTCTTGAGG
AtKUP5_F	TGTATCTACTGGGACATGGG
AtKUP5_R	CGTTCCGCATACACATTTGA
AtKUP6_F	TTATATGAAAGCGAAGCCAGG
AtKUP6_R	GCCTCACGCTTCTACTTT
AtKUP7_F	CACAAAGCGAAAGAATCAGG
AtKUP7_R	GTCGGTATGACATACATGGT
AtKUP8_F	CTCGTGAGGCAGGAATG
AtKUP8_R	GGTCTCTCTACTCCTCACG
AtKUP9_F	AGGAGGAGGAGACGGATGAG
AtKUP9_R	CTTGCTAAGATTTGTAGGGC
AtKUP10_F	GAAGTGGAGTTCATAAACGGG
AtKUP10_R	GCCATCTTCAATGTTCCTCA
AtKUP11_F	GTACACATAATGGGGAACACG
AtKUP11_R	GAGAGCCTTTTGAATGTTGG
AtKUP12_F	GAGAGAAGCCACAGACTCG
AtKUP12_R	GCTGCGAATCTCACTGTT
SKOR_F	TCAAATCTCTTCGTTTCCG
SKOR_R	CGGAGGCTTCTTGTGTATTA
At ROC3_F	ATCGTGATGGAGCTTTACGC
At ROC3_R	TCGGTGAAAGCTTGATCCTT
